# Divergent evolutionary behavior of H3 histone gene and rDNA clusters in venerid clams

**DOI:** 10.1186/s13039-015-0150-7

**Published:** 2015-06-24

**Authors:** Daniel García-Souto, Concepción Pérez-García, Paloma Morán, Juan J. Pasantes

**Affiliations:** Departamento Bioquímica, Xenética e Inmunoloxía, Universidade de Vigo, E-36310 Vigo, Spain

**Keywords:** Venerid clams, Chromosome, Fluorescent *in situ* hybridization, Histone genes, Ribosomal RNA genes

## Abstract

**Background:**

Histone H3 gene clusters have been described as highly conserved chromosomal markers in invertebrates. Surprisingly, in bivalves remarkable interspecific differences were found among the eight mussels and between the two clams in which histone H3 gene clusters have already been located. Although the family Veneridae comprises 10 % of the species of marine bivalves, their chromosomes are poorly studied. The clams belonging to this family present 2n = 38 chromosomes and similar karyotypes showing chromosome pairs gradually decreasing in length. In order to assess the evolutionary behavior of histone and rRNA multigene families in bivalves, we mapped histone H3 and ribosomal RNA probes to chromosomes of ten species of venerid clams.

**Results:**

In contrast with the reported conservation of histone H3 gene clusters and their intercalary location in invertebrates, these loci varied in number and were mostly subterminal in venerid clams. On the other hand, while a single 45S rDNA cluster, highly variable in location, was found in these organisms, 5S rDNA clusters showed interspecific differences in both number and location. The distribution patterns of these sequences were species-specific and mapped to different chromosomal positions in all clams but *Ruditapes decussatus*, in which one of the minor rDNA clusters and the major rDNA cluster co-located.

**Conclusion:**

The diversity in the distribution patterns of histone H3 gene, 5S rDNA and 28S rDNA clusters found in venerid clams, together with their different evolutionary behaviors in other invertebrate taxa, strongly suggest that the control of the spreading of these multigene families in a group of organisms relies upon a combination of evolutionary forces that operate differently depending not only on the specific multigene family but also on the particular taxa. Our data also showed that H3 histone gene and rDNA clusters are useful landmarks to integrate nex-generation sequencing (NGS) and evolutionary genomic data in non-model species.

## Background

The analysis of the chromosome changes encompassing the evolution of a group of organisms relies on the accurate identification of their chromosomes. When chromosome-specific painting probes are not available, as frequently happens in invertebrates, and karyotypes are composed by chromosomes gradually decreasing in length, chromosomal identification turns into a very difficult task. In those cases, the hybridization of highly conserved repetitive sequences, among which ribosomal RNA (rRNA) and histone genes are paramount, usually constitutes a first step in finding chromosome-specific probes.

Eukaryotic genomes present multiple copies of genes encoding histones, the basic proteins responsible of packaging DNA into chromatin. The histone multigene family includes five main types of genes, those encoding the histones of the nucleosome core particle (H2A, H2B, H3 and H4) and those for the linker histones (H1) [1]. rRNA genes are also organized in multigene families, one expressing for the 18S, 5.8S and 28S rRNAs (45S rDNA) and the other for the 5S rRNA [2]. Histone and rRNA genes in invertebrates are usually organized in tandem arrays clustered in one or more chromosomal positions, although other organizations have also been described [3, 4]. The evolutionary dynamics of both histone gene and rDNA clusters has been analyzed in only a few groups of these organisms, i.e. grasshoppers [5, 6], beetles [7, 8], aphids [9] and moths [10]. Whereas histone gene clusters were extremely conserved in number and location in all these groups, 45S and 5S rDNAs showed high degrees of variation.

In bivalves, the genomic organization of the histone genes has been studied using molecular methodologies in species belonging to the families Mytilidae [11–14], Pectinidae [15] and Veneridae [16], showing, in all of them, a tandemly arranged organization. Usually, the clusters comprise only core histone genes but repeated clusters including linker histone and/or other genes have also been reported. In these organisms histone H3 genes have been mapped to chromosomes in only eight mussels [13, 14, 17–20], four scallops [21], one oyster [22] and two clams [23]. In comparison with 45S and 5S rDNA clusters, surprisingly remarkable differences in number and location of the histone H3 gene clusters were found, more outstandingly among mussels and clams.

The venerid clams of the family Veneridae (Rafinesque 1815) represent almost 10 % of the species of marine bivalves [24]. Phylogenetic relationships among the species of this family were the subject of many recent investigations using DNA sequences whose results, in some cases, challenged the traditional, morphologically based classification [25–29]. In contrast, the chromosomal characterization of the venerid clams lags far beyond the knowledge achieved for other bivalve families. Classical venerid cytogenetics was limited to determine chromosome numbers and karyotypes in a few species [30–32]. More recently, a restriction endonuclease banding pattern was described in *Ruditapes decussatus* [33] and some repetitive DNA sequences were mapped by fluorescent *in situ* hybridization (FISH). The location of telomeric sequences and/or major and minor rDNA was reported for *Mercenaria mercenaria* [34, 35], *Dosinia exoleta* [36, 37], *Ruditapes decussatus* and *Ruditapes philippinarum* [37, 38], *Polititapes aureus* and *Polititapes rhomboides* [23] and *Venerupis corrugata* and *Venus verrucosa* [37]. On the other hand, histone gene clusters, as indicated above, were only mapped to chromosomes of *Polititapes aureus* and *Polititapes rhomboides* [23].

In order to get a better understanding of the evolutionary behavior of these multigene families in these organisms, we have hybridized H3 gene, 5S rDNA and 28S rDNA probes to mitotic and meiotic chromosomes of ten species of clams of the family Veneridae, *Ruditapes philippinarum*, *Ruditapes decussatus*, *Venerupis corrugata*, *Clausinella fasciata, Chamelea gallina*, *Venus verrucosa*, *Venus casina*, *Dosinia exoleta*, *Dosinia lupinus* and *Petricola litophaga*.

## Results

**Fig. 1:**
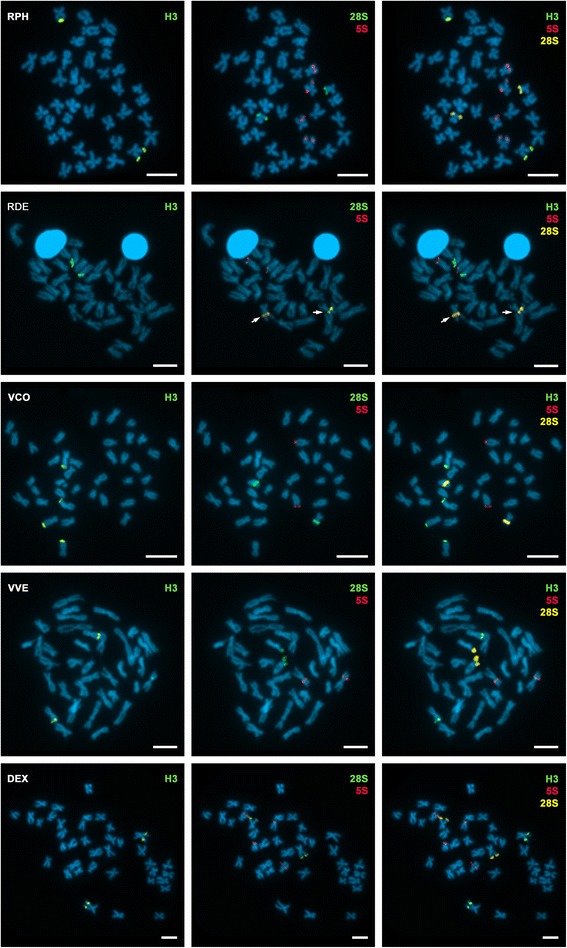
Chromosomal location of H3 histone genes in venerid clams. H3 histone gene (H3) probes mapped by FISH to mitotic chomosomes of *Ruditapes philippinarum* (RPH), *Ruditapes decussatus* (RDE), *Venerupis corrugata* (VCO), *Venus verrucosa* (VVE) and *Dosinia exoleta* (DEX). To ascertain the chromosomal position of core histone gene clusters in relation to rDNA clusters, the same metaphases were rehybridized using 5S rDNA (5S) and major rDNA (28S) probes. Excluding 5S and major rDNA in RDE (arrows), all signals are on different chromosome pairs. Scale bars, 5 μm

**Fig. 2:**
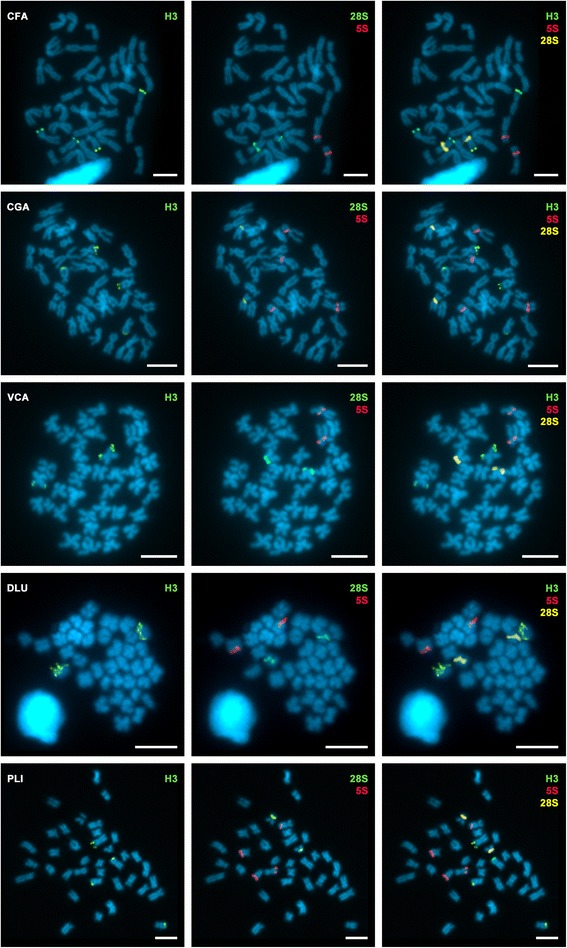
Chromosomal location of H3 histone genes (H3), 5S rDNA (5S) and major rDNA (28S) in venerid clams. Single FISH using H3 histone gene probes mapped to chromosomes of *Clausinella fasciata* (CFA), *Chamelea gallina* (CGA), *Venus casina* (VCA), *Dosinia lupinus* (DLU), and *Petricola litophaga* (PLI), followed by double-FISH using 5S rDNA (5S) and major rDNA (28S) probes on the same metaphase plates. All signals for the different probes appear at different chromosome pairs with the exception of H3 histone gene and major rDNA in *Clausinella fasciata* (CFA, first row). Scale bars, 5 μm

**Fig. 3:**
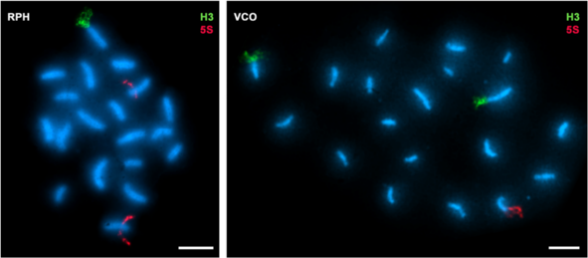
Subterminal H3 histone gene clusters in venerid clams. Examples of FISH to surface spread synaptonemal complexes of *Ruditapes philippinarum* (RPH) and *Venerupis corrugata* (VCO) clearly denote the subterminal location of the H3 histone gene clusters (H3, green). 5S rDNA clusters (5S, red) are also subterminal in VCO but intercalary in RPH. Scale bars, 5 μm

FISH experiments identified a total of 14 *loci* for the histone H3 gene in the 10 species analyzed (Figs. 1 and 2). We detected a single core histone gene cluster in six of the species and two clusters in the remaining four. Regarding their chromosomal location, 11 of the histone clusters were close to the telomeres, two were intercalary, and the remaining one close to the centromere. The subterminal location of the clusters was confirmed by FISH on synaptonemal complex spreads (Fig. 3). A summary of the data obtained in this work, together with the other currently available FISH mapping data for the family Veneridae, is presented in Table 1. The species were arranged following the molecular phylogenetic tree suggested by [29] (Chen et al.) and assigned both to their proposed clade groups (A1, A2, A3, A4, B1) and the subfamilies of the traditional classification (Tapetinae, Chioninae, Venerinae, Dosininae and Petricolinae). Histone H3 gene clusters mapped to a single locus in two of the three analyzed species included in clade A1 (Tapetinae: *Ruditapes philippinarum* and *Ruditapes decussatus*), to two of the four species in clade A2 (Venerinae: *Venus verrucosa* and *Venus casina*), and in the two species in clade A4 (Dosininae: *Dosinia exoleta* and *Dosinia lupinus*). In the remaining four species, one in clade A1 (Tapetinae: *Venerupis corrugata*), two in clade A2 (Chioninae: *Clausinella fasciata* and *Chamelea gallina*), and one in clade B1 (Petricolinae: *Petricola litophaga*), core histone gene clusters mapped to two loci situated in different chromosome pairs.

**Table 1:** Chromosomal location of core histone gene and rDNA clusters in venerid clams

Clade	Subfamily	Species	Histone genes	Major rDNA	5S rDNA	References
A1	Tapetinae	*Ruditapes philippinarum*	4q ter (m)	8p ter (m)	5q ic (st)	[29, 30] this study
					6q ic (st)	
		*Ruditapes decussatus*	4p cen (st)	3q ic (sm)	3q ic (sm)	[29, 30] this study
					8q ter (st)	
		*Polititapes aureus*	2p ter (m)	5p ic (m)	17q ter (st)	[15]
			2q ter (m)			
			3q ter (m)			
			8q ter (m)			
		*Venerupis corrugata*	2q ter (m)	10q ic (m)	9q ter (st)	[29] this study
			4q ter (sm)			
		*Polititapes rhomboides*	5q ic (m)	17q ter (st)	9p ter (m)	[15]
			12q cen (st)			
A2	Chioninae	*Clausinella fasciata*	10q ter (m)	10q cen (sm)	5p ic (sm)	this study
			12p ter (sm)			
		*Chamelea gallina*	15q ter (m)	13p cen (sm)	5p ic (sm)	this study
			18q ter (st)		9q ic (m)	
	Venerinae	*Venus verrucosa*	13q ter (m)	12p ter (sm)	9q ic (m)	[29] this study
		*Venus casina*	9q ter (m)	16q ter (st)	6p ic (sm)	this study
A3	Chioninae	*Mercenaria mercenaria*	unknown	10q ic (sm)	unknown	[27]
				12p ter (st)		
A4	Dosininae	*Dosinia exoleta*	2q ic (m)	3q ter (m)	13q ic (sm)	[28, 29] this study
					15p ter (sm)	
		*Dosinia lupinus*	9q ic (m)	12q ic (m)	14q ter (sm)	this study
B1	Petricolinae	*Petricola litophaga*	3p ter (m)	19p cen (m)	5q ic (m)	this study
			14q ter (m)		12q ic (st)	
					12q ic (st)	

*p* short arm, *q* long arm

*cen* subcentromeric, *ic* intercalar, *ter* subterminal

*(m)* metacentric, *(sm)* submetacentric, *(st)* subtelocentric

**Fig. 4:**
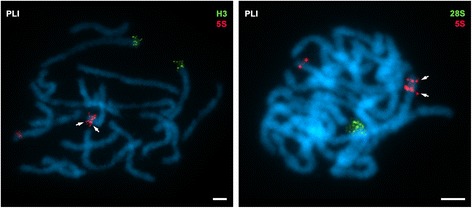
5S rDNA clusters on *Petricola litophaga*. FISH of H3 histone gene (H3), 5S (5S) and 28S (28S) rDNA probes to prophase I meiotic bivalents of *Petricola litophaga* (PLI) clearly show the presence of two distinct 5S rDNA signals (arrows) on a single bivalent. Scale bars, 5 μm

In order to investigate the location of the core histone gene clusters in relation to rDNAs, we performed double and sequential FISH experiments using core histone gene, major rDNA and 5S rDNA probes in the five species of clams in which the location of rDNA sequences was already known (Fig. 1) and in the other five in which not previous data were available (Fig. 2). Whereas all species presented a single major rDNA cluster per haploid genome, the number of 5S rDNA clusters was one in five of the species (*Venerupis corrugata*, *Clausinella fasciata, Venus verrucosa*, *Venus casina* and *Dosinia lupinus*), two in four (*Ruditapes philippinarum*, *Ruditapes decussatus*, *Chamelea gallina* and *Dosinia exoleta*) and three in the remaining one, *Petricola litophaga* (Table 1). Taking into account that two of the three 5S rDNA clusters detected in *Petricola litophaga* were close together, sometimes giving double FISH signals and other times a single signal, we performed FISH on prophase I meiotic bivalents to clarify the true nature of the signals. As shown in Fig. 4, three clearly different signals were detected, two of them on the same bivalent.

*Clausinella fasciata* was the only species in which a single chromosome harbors both histone gene and rDNA cluster; in this species, a subterminal histone gene cluster and a subcentromeric major rDNA cluster appeared in the long arm of chromosome 10 (Fig. 2). Confirming previously published results, in *Ruditapes decussatus* the signals for one of the 5S rDNA clusters and the major rDNA cluster overlap on chromosome 3 (Fig. 1).

**Fig. 5:**
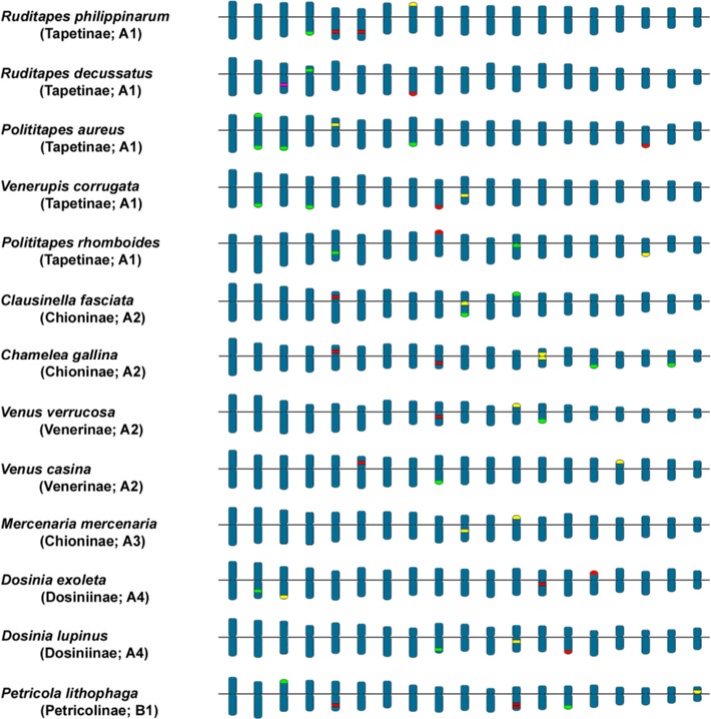
Ideograms showing the chromosomal location of H3 histone genes, 5S rDNA and major rDNA in thirteen species of Veneridae. The green areas represent the H3 histone gene clusters, the red areas the 5S rDNA clusters and the yellow areas the major rDNA clusters. The magenta area in *Ruditapes decussatus* indicates overlapping of major and 5S rDNA signals

Figure 5 presents an ideogrammatic representation of the karyotypes of the 13 species of Veneridae for which histone gene and/or rDNA mapping results are currently available.

FISH experiments using a vertebrate telomeric (C_3_TA_2_)_3_ peptide nucleic acid (PNA) probe gave terminal signals at the ends of the sister chromatids of every mitotic chromosome in all clam species. No intercalary signals were detected.

## Discussion

Multigene families are useful cytogenetic markers not only for studying chromosomal evolution but also for the correct interpretation of the data obtained via NGS. Aligning and assembling NGS data is a hard task in many non-model organisms mainly due to the obstacles posed by the abundance of repetitive DNA sequences. The physical location of repetitive gene families will help in this task.

In this work we have demonstrated the presence of remarkable interspecific differences in the physical location of H3 histone gene clusters in venerid clams. These clusters have been described as highly conserved chromosomal markers in other invertebrate groups. Our data, together with previously published results [23], showed variation for the number of core histone gene sites in venerid clams, with six species carrying a single cluster, five presenting two clusters and one showing four clusters (Table 1). The observed variation in the number of core histone gene clusters did not present any obvious relationship with the currently taxonomic classification of the family Veneridae or its most represented clades. In clade A1 (Tapetinae) there were species showing one (*Ruditapes philippinarum* and *Ruditapes decussatus*), two (*Venerupis corrugata* and *Polititapes rhomboides*) and four (*Polititapes aureus*) histone gene clusters. A similar divergence also applied to clade A2, including species of the subfamilies Venerinae and Chioninae, in which two species (*Venus verrucosa* and *Venus casina*) showed a single cluster and the other two species (*Clausinella fasciata* and *Chamelea gallina*) presented two clusters.

These differences in the number of core histone gene clusters in related species are coincident with results reported for other bivalve families. While three species of scallops and one mussel showed a single core histone gene cluster [17, 21], one scallop and six mussels presented two [17, 18, 20, 21] and one mussel had four [19]. This behavior differs with those described for other invertebrate groups in which histone gene clusters have been reported to be a highly conservative cytogenetic marker [5–10].

In contrast with the variability in number, 75 % of the H3 histone gene clusters detected in venerid clams (15 of a total of 20) were located at subterminal chromosome positions. This is unusual for bivalves and also for other invertebrates; in fact, barely a 30 % of the core histone gene clusters detected in mussels of the family Mytilidae (5 of 17) [17–20] and only a 40 % of those reported in the scallops of the family Pectinidae (2 of 5) [21] were subterminal. For other invertebrates, the subterminal position of the histone gene signal has only been described in three grasshoppers [5].

Taking into account the above mentioned data, the presumably ancestral situation in venerids is a single subterminal core histone gene cluster. Although the mechanisms that allowed increasing the number of clusters remain to be determined, the presence of most of these sequences in close proximity to the telomeres might facilitate their spreading to non-homologous chromosomes. Subtelomeric chromosomal regions are characterized in many eukaryotes by accumulating repeat sequences and harbor many breakpoints [39, 40]. These features, together with the telomere clustering in meiotic cells, probably favors their implication in sequence exchanges between non-homologous chromosomes and contribute to their highly dynamic behavior [41].

Regarding rDNA, the clams of the family Veneridae showed variation in both number and chromosomal location of the 5S rDNA clusters but only in location of the 45S rDNA clusters. Whereas all species had a single 45S rDNA cluster, the number of 5S rDNA clusters varied; seven species showed a single cluster, six species two and *Petricola litophaga* three. The conservation in the number of major rDNA clusters in Veneridae was not paired by their chromosomal location; subterminal, intercalary and subcentromeric locations were found. The 5S rDNA clusters were either subterminal or intercalary. As happened with H3 histone gene clusters, the variant 45S and 5S rDNA arrangements did not concord with the taxonomic distribution of the species of the family Veneridae; species belonging to the same clade or subfamily showed 45S and 5S rDNA clusters differing in number and chromosome location (Table 1).

These results partially differ with those found in other bivalve families. While mytilid mussels showed one to four major rDNA and two to five 5S rDNA clusters [17–20, 42], both Pectinidae and Ostreidae species presented one or two major and 5S rDNA clusters [31, 43–47].

The evolutionary dynamics of the major rDNA clusters in venerid clams is similar to the one reported for tortricid moths [10] but the opposite to the described in other invertebrate groups in which both the number and the position of these sequences has been reported as highly variable [5–8]. In contrast, the behavior of the 5S rDNA is common to some other invertebrate taxa in which it has been described as a highly variable chromosomal marker [5–8] whose movement has been attributed to transposition and/or unequal crossover [48].

In conclusion, the diversity in the distribution patterns of histone H3 gene, 5S rDNA and 28S rDNA clusters found in venerid clams, together with their different evolutionary behaviors in other invertebrate taxa, strongly suggest that the control of the spreading of these multigene families in a group of organisms relies upon a combination of evolutionary forces that operate differently depending not only on the specific multigene family but also on the particular taxa. On the other hand, our data clearly showed that the number and position of the H3 histone gene and rDNA clusters are species-specific in venerid clams and that the complexity of their evolutionary patterns make them useful landmarks that can contribute to integrate NGS and evolutionary genomic data in non model species.

## Methods

### Venus clam specimens

Samples of the Japanese carpet shell *Ruditapes philippinarum* (Adams and Reeve 1850), the grooved carpet shell *Ruditapes decussatus* (Linnaeus 1758), the pullet carpet shell *Venerupis corrugata* (*pullastra*) (Gmelin 1791), the banded venus *Clausinella fasciata* (da Costa 1778), the warty venus *Venus verrucosa* (Linnaeus 1758), the pale venus *Venus casina* (Linnaeus 1758), the rayed artemis *Dosinia exoleta* (Linnaeus 1758), the smooth artemis *Dosinia lupinus* (Linnaeus 1758), and the boring petricola *Petricola litophaga* (Retzius1788) were collected from natural and cultured populations in Ría de Pontevedra and Ría de Vigo (NW Spain). Samples of the striped venus *Chamelea gallina* (Linnaeus 1758) were collected from natural populations in the Gulf of Valencia (E Spain). The nomenclature used for these taxa follows the World Register of Marine Species database [49].

### Chromosome preparation

Mitotic metaphase and meiotic prophase I spreads were prepared as previously described [50]. In brief, after exposing the clams to colchicine (0.005 %, 12 h), gills and gonads were removed. The tissues were treated with diluted sea water (50 %, 25 %, 1 h) and fixed in ethanol/acetic acid (3:1, 1 h). The cell suspension obtained after dissociating the tissue (60 % acetic acid) was dropped onto heated slides.

Synaptonemal complexes were spread as indicated by Hurtado and Pasantes [36]. Suspensions of male gonadic cells were spread on slides using 0.1 M sucrose and 0.5 % Triton X-100, fixed with paraformaldehyde (4 %), rinsed in distilled water and air-dried.

### DNA extraction, PCR amplification and probe labeling

DNA was extracted using the method published by Winnepenninckx et al. [51]. The tissue was homogenized in hexadecyltrimethylammoniumbromide (CTAB) buffer and digested with pronase (1.5 mg/mL, 60 °C). The DNA was extracted with chloroform/isoamyl alcohol (24/1).

FISH probes were amplified by polymerase chain reaction (PCR). Reactions used 50 ng DNA, 1x PCR buffer, 0.5 mM each dNTP, 2.5 mM MgCl_2_, 1 μM each primer and 1 U BIOTAQ DNA polymerase (Bioline) in a volume of 20 μl. A fragment of the 28S rRNA gene of the major rDNA repeat was amplified using universal primers [52]. Primers designed from the sequence of the 5S rRNA of *M. edulis* [53] were used to amplify the whole repeat of the 5S rDNA. The amplification of the histone H3 genes used primers described by Giribet and Distel [54].

After an initial denaturation at 95 °C, 30 cycles (95 °C, 20 s; 48 °C, 20 s; 72 °C, 30 s) of amplification and a final extension step of 7 min at 72 °C were applied in a GeneAmp PCR system 9700 (Applied Biosystems). Electrophoresis on 2 % agarose gels demonstrated that single PCR products were obtained. 28S rDNA probes were labeled by nick translation (Roche Applied Science) with biotin-16-dUTP (Roche Applied Science) and/or digoxigenin-11-dUTP (10x DIG Labeling Mix, Roche Applied Science). Histone H3 gene and 5S rDNA probes were labeled by PCR either with biotin-16-dUTP (20 μM) or digoxigenin-11-dUTP (5 μM). The labeled PCR products were precipitated before FISH.

### Fluorescent *in situ* hybridization (FISH)

Single and double FISH experiments using biotin and digoxigenin labeled histone H3 gene and 28S and 5S rDNA probes were performed following previously published methods [19, 37]. Slides were pre-treated with RNase and pepsin before denaturating them for 2 min at 70 °C (mitotic chromosomes) or 80 °C (meiotic chromosomes). Hybridizations were performed overnight at 37 °C. Signal detection was carried out with fluorescein avidin and biotinylated anti-avidin for the biotinylated probes and with mouse anti-digoxigenin and anti-mouse TRITC for the probes labeled with digoxigenin. Slides were counterstained for 8 min with 4′-6-diamidin-2-fenilindol (DAPI: 0.14 μg mL^−1^ in 2xSSC) and mounted in antifade (Vectashield, Vector). In addition we also performed FISH with a vertebrate telomeric (C_3_TA_2_)_3_ peptide nucleic acid (PNA) probe (Applied Biosystems) following the protocol indicated by the supplier.

A Nikon Eclipse-800 microscope equipped with an epifluorescence system was used to record a minimum of 20 metaphase plates per probe or combination of probes in 10 specimens (5 male, 5 female) per species. Separated images for each fluorochrome were obtained with a DS-Qi1Mc CCD camera (Nikon) controlled by the NIS-Elements software (Nikon). Merging of the images was performed with Adobe Photoshop.

For each species, karyotypes were constructed from 10 complete metaphase plates showing FISH signals. Relative lengths and centromeric indices were determined. Chromosomes nomenclature follows Levan et al. [55].

## Abbreviations

CFA: *Clausinella fasciata*
CGA: *Chamelea gallina*
DAPI: 4′,6-diamidino-2-phenylindole
DEX: *Dosinia exoleta*
DLU: *Dosinia lupinus*
FISH: Fluorescence *in situ* hybridization
NGS: Next-generation sequencing
PCR: Polymerase chain reaction
PLI: *Petricola litophaga*
PNA probe: Peptide nucleic acid probe
RDE: *Ruditapes decussatus*
rDNA: Ribosomal DNA
RPH: *Ruditapes philippinarum*
rRNA: Ribosomal RNA
TRITC: Tetramethyl rhodamine isothiocyanate
VCA: *Venus casina*
VCO: *Venerupis corrugata*
VVE: *Venus verrucosa*
